# Depression, Anxiety and Quality of Life in Staff of a Hospital in Athens: A Study in the Aftermath of the Debt Crisis Era

**DOI:** 10.15388/Amed.2021.28.2.3

**Published:** 2021-07-29

**Authors:** Despoina Melemeni, Konstantinos Mantzouranis, Vasiliki Epameinondas Georgakopoulou, Kyriakos Tarantinos, Nikolaos Garmpis, Christos Damaskos, Pagona Sklapani, Serafeim Chlapoutakis, Nikolaos Trakas, Xanthi Tsiafaki, Ioanna V. Papathanasiou

**Affiliations:** 1^st^ Pulmonology Department Sismanogleio Hospital, Athens, Greece; 1^st^ Pulmonology Department Sismanogleio Hospital, Athens, Greece; Pulmonology Department, Laiko General Hospital, Athens, Greece; 1^st^ Pulmonology Department Sismanogleio Hospital, Athens, Greece; Second Department of Propedeutic Surgery, Laiko General Hospital, Medical School, National and Kapodistrian University of Athens, Athens, Greece; N.S. Christeas Laboratory of Experimental Surgery and Surgical Research, Medical School, National and Kapodistrian University of Athens, Athens, Greece; Department of Cytology, Mitera Hospital, Athens, Greece; Department of Thoracic Surgery, Agios Savvas Hospital, Athens, Greece; Department of Biochemistry, Sismanogleio Hospital, Athens, Greece; 1^st^ Pulmonology Department Sismanogleio Hospital, Athens, Greece; Faculty of Nursing, University of Thessaly, Larissa, Greece Hellenic Open University, Patras, Greece

**Keywords:** hospital staff, mental health, well-being, depression, quality of life, anxiety

## Abstract

**Background:**

Several studies investigated the mental health needs of hospital staff in Greece during the debt crisis era. Yet, no relevant data are available regarding the mental health of hospital staff after this period. The aims of this study are: 1) To investigate the prevalence of clinically significant depression and anxiety in healthcare workers in a general hospital in Athens, Greece; 2) to search for the association of quality of life with anxiety and depression in those workers; 3) to investigate the association of sociodemographic characteristics with those parameters.

**Methods:**

The Zung Depression Rating Scale, the Zung Anxiety Rating Scale, the Short-Form Survey-12, assessing quality of life, and sociodemographic assessments were administrated in 110 workers of a public hospital in Athens, Greece. The assessments were completed during January, 2020.

**Results:**

Of the study participants, 38.2% had clinically significant anxiety and 6.4% had clinically signifi-cant depression. Males had lower scores of depression compared to females (p=0.003). As for the effects of educational level, differences were noted in psychological quality of life between secondary education participants when compared to tertiary education (Mean Difference -3.527, p=0.021), post-graduate (Mean Difference -3.937, p=0.012) and PhD participants (Mean Difference -5.100, p=0.007). Quality of life and its psychological and physical health subscales had strong inverse associations with depression and anxiety (p=0.000).

**Conclusions:**

Relevant interventions are necessary to decrease anxiety in hospital staff, which is elevated in the aftermath of the debt crisis period. In addition, health policy makers have to reduce the gender gap in mental health between male and female workers, since the latter had higher levels of depression.

## Abstract (lt)

**Depresijos ir nerimo paplitimas tarp Atėnų ligoninės darbuotojų ir jų gyvenimo kokybė. Tyrimas pasibaigus skolų krizei:**

**Santrauka. *****Problema.*** Keletoje tyrimų nagrinėta Graikijos ligoninių darbuotojų psichikos sveikata skolų krizės metu. Tačiau aktualių duomenų apie ligoninių darbuotojų psichikos sveikatą pasibaigus šiam laikotarpiui nėra. Šio tyrimo tikslai: 1) ištirti kliniškai svarbios depresijos ir nerimo paplitimą tarp sveikatos priežiūros darbuotojų Atėnų, Graikija, ligoninėje; 2) nustatyti tų darbuotojų gyvenimo kokybės ir nerimo ar depresijos ryšį; 3) išnagrinėti socialinių ir demografinių veiksnių bei tų parametrų ryšį.

***Metodai.*** Zungo depresijos vertinimo skalė, Zungo nerimo vertinimo skalė, Gyvenimo kokybės klausimynas SF-12 buvo naudojami 110 Atėnų viešosios ligoninės darbuotojų apklausai, taip pat įvertinti socialinius ir demografinius veiksnius. Vertinimas atliktas 2020 m. sausio mėnesį.

***Rezultatai.*** 38,2 proc. tyrimo dalyvių pasireiškė kliniškai svarbus nerimas, o 6,4 proc. – kliniškai svarbi depresija. Vyrams, palyginti su moterimis (p = 0,003), depresija pasireiškė rečiau. Išsilavinimas taip pat lėmė psichologinės gyvenimo kokybės skirtumus tarp dalyvių, turinčių vidurinį išsilavinimą, palyginti su turinčiais aukštąjį išsilavinimą (vidurkių skirtumas – 3,527, p = 0,021), magistrantais (vidurkių skirtumas – 3,937, p = 0,012) ir doktorantais (vidurkių skirtumas – 5,100, p = 0,007). Santykis tarp gyvenimo kokybės skalės, jos psichologinės ir fizinės sveikatos poskalių buvo atvirkštinis depresijai ir nerimui (p = 0,000).

***Išvados.*** Norint sumažinti ligoninės darbuotojų nerimo lygį, padidėjusį po skolų krizės, būtina taikyti intervencines priemones. Be to, sveikatos politikos formuotojai turi sumažinti atskirtį tarp vyrų ir moterų psichikos sveikatos, nes pastarųjų depresijos lygis buvo aukštesnis.

**Raktažodžiai: **ligoninės darbuotojai, psichikos sveikata, gerovė, depresija, gyvenimo kokybė, nerimas

## Introduction

In Greece, prior to the debt crisis era (2008-2018), hospital staff had low levels of depression, but considerable levels of anxiety. Tselebis et al. investigated the levels of anxiety and depression in physicians and nurses [1]. As they found, only 1% of males and 4% of female physicians, as well as 2% of male and 3% of female nurses had scores above the 1st quartile in the Beck-Depression Inventory. As for anxiety, 29% of male and 37% of female physicians, as well as 30% of male and 34.5% of female nurses had scores above the 1st quartile in the Spielberger State-Trait Anxiety Inventory. Thus, prior to the debt crisis the Greek hospital staff had high levels of anxiety, but low levels of depression.

Debt crisis has affected the people and the healthcare systems of the countries that suffered due to the increased rates of unemployment, the loss of health insurance, the decreased income and the inability of individuals to bear the healthcare costs, and the poor health outcomes leading to in increased morbidity and mortality [2]. At the beginning of the crisis, mental health experts warned about the necessity of measures to protect mental health [3]. According to two studies, in Greece, the suicide rate increased, both in males and females of working age, by 56% between 2007 and 2011 and by 35% between 2010 and 2012 due to the increased rates of unemployment [4,5]. In addition, patients suffering from severe psychiatric disorders have been among the first to be affected from the current economic crisis [6]. Furthermore, according to data available from the Ministry of Health, Directorate for Mental Health, visits to emergency and outpatient departments and mental health facilities in the National Healthcare System increased to 120% between 2011 and 2013. These were significantly correlated with unemployment and low income [7]. 

Several studies tried to investigate the mental health needs of health professionals during the debt crisis era. Tsaras et al. investigated the prevalence of depression and anxiety in mental health nurses (N=110), finding that 52.7% and 48.2%, respectively, met the cutoff of 3 for depression and anxiety of PHQ-2 and GAD-2 scales [8]. In another study in mental health professionals, from several mental units in Greece, was found that 12.20% and 9.90% had clinically significant anxiety and depression, respectively [9]. In an additional study in oncology nurses, Karanikola et al. found that 11% of the study participants met the criteria for clinically significant anxiety [10]. Furthermore, the levels of anxiety in nurses during the debt crisis era were found to be higher compared to the general population of the country [11].

The aforementioned evidence indicates that prior to the debt crisis the experience of anxiety was common in hospital staff, but the experience of depression was not. The major difference in the debt crisis era was that, apart from the methodological differences in each of the studies, considerable proportions of depression were also reported. To date, hospital staff has to encounter the COVID-19 pandemic, which leads to several aggravating effects for the mental health and quality of life of hospital staff [12,13]. Although relevant data from Greece are yet limited [14], it can be expected that the pandemic will have a negative effect on mental health and quality of life of the hospital staff in Greece. Thus, this small period after the debt crisis and prior to the COVID-19 pandemic is worthy of investigation, since it could reflect the mental health needs of the Greek hospital staff in normal circumstances. It could also serve as a reference for future studies trying to depict the effect of the pandemic on hospital staff’s mental health and quality of life, since relevant evidence is necessary to make comparisons.

The aims of the present study were to investigate the prevalence of clinically significant depression and anxiety in healthcare workers in a general hospital in Athens, Greece, the association of quality of life with anxiety and depression in those workers and the association of sociodemographic characteristics with those parameters.

## Materials and Methods

### Study design

The design of the present study was cross-sectional. The data collection took place at “Sismanogleio General Hospital” during January 2020. The study gained approval by the relevant Institutional Board (protocol number 7079/7-4-20). The study was in line with the declaration of Helsinki in 1995 (as revised in Edinburgh 2000).

### Participants

Inclusion criteria set for study participation were: 1) being medical staff (doctors, nurses, allied health professionals) and 2) working in our hospital for the time interval between January 2008 and January 2020.

### Sociodemographic data

The sociodemographic data of the present study were the following: 1) gender (male, female), 2) age, 3) years in service, 4) educational status (primary, secondary, vocational training, tertiary, postgraduate, Doctor of Philosophy (PhD), 5) occupational status, 6) family status (unmarried, married, divorced, widowed), 7) number of children.

### Depression

The participants’ depression levels were assessed by the use of the Zung-Depression Rating Scale (ZDRS). This self-reported instrument includes 20 items scored in a 1-4 Likert type scale. Higher scores indicate higher levels of depression [15]. The Greek version of the ZDRS was used in this study, upon approval from the research team which translated and validated the instrument in Greek [16]. The Greek version of the ZDRS also consists of 20 items concerning affective, psychological, and somatic symptoms. The patient mentions the frequency with which the symptom is experienced (that is: a little = 1, some = 2, a good part of the time = 3, or most of the time = 4). A total score is derived by summing the responder item scores, ranging from 20 to 80. A score of 49 and above indicates abnormal symptoms of depression, while a score of 70 and above indicates severe depression (50-59 mild, 60-69 moderate, 70 and above severe depression) [16].

### Anxiety

The responders’ anxiety levels were measured by the use of the Zung-Anxiety Rating Scale (ZARS). This self-reported instrument assesses the responders’ levels of anxiety by the use of 20 items scored on a 1–-4 Likert type scale. Higher scores indicate higher levels of anxiety [17]. The Greek version of the ZARS was used in the present study, after gaining the relevant approval from the authors that translated and validated this instrument in Greek [18]. The Greek version of the ZARS is also based on scoring in four groups of manifestations: cognitive, autonomic, motor and central nervous system symptoms, using 20 items scored on a 1–4 Likert type scale. The scoring is based on these responses: “a little of the time,” “some of the time,” “good part of the time,” “most of the time.” A total score is derived by summing the responder item scores, ranging from 20 to 80. A score of 45 and above indicates abnormal symptoms of anxiety, while a score of 60 and above indicates marked to severe anxiety (4559 mild to moderate, 60-74 marked to severe, 75 and above extreme anxiety levels) [18]. 

### Quality of life

The responders’ quality of life was assessed by the use of Short-Form Survey-12 (SF-12). The SF-12 assesses a wide range of quality of life parameters through 12 related questions. Two separate sub-scales are calculated from this instrument, which refer to physical and psychological quality of life [19]. The Greek version of the SF-12 was used in the present study, upon relevant approval from the developers of this version [20]. The Greek version of the SF-12 also contains the physical health-related domains: General Health (GH), Physical Functioning (PF), Role Physical (RP), and Body Pain (BP); and mental health-related scales: Vitality (VT), Social Functioning (SF), Role Emotional (RE), and Mental Health (MH) [20]. 

### Procedures

Totally, the assessments of the present study were completed by 110 responders. More specifically, the potential participants were approached face to face and informed of the study purpose. Prior to study inclusion, the participants provided their written consent. The assessments were completed instantly or returned at another mutually agreed time, in case participants desired so. The recruited process took place during January 2020. At next, the data were entered in the SPSS statistical soft-ware in order to be analyzed.

### Statistical analysis

At first, descriptive statistics were applied in order to calculate the demographic characteristics of the participants. The analysis was carried out by the use of absolute values and proportions, in case the variables were categorical, and by the use of mean values and standard deviations, in case the variables were numerical. In addition, descriptive analysis was applied to calculate the proportion of responders that were above the cutoff value of clinically significant anxiety and depression. At next, normality tests were carried out to investigate whether parametric or nonparametric analyses had to be applied. The Kolmogorov–Smirnov Test was not violated for depression (p=0.172), while it was violated for anxiety (p=0.003) and quality of life (p=0.001). Thus, in all the analysis involving only depression, parametric tests were applied, while in all the other analysis nonparametric tests were applied. The associations of the responders’ demographic variables with the scores of the instruments were analyzed by Independent Samples T-test and Mann–Whitney U test, in case the independent variables were categorical with two values, and by the use of One-Way ANOVA and Kruskal–Wallis, in case the categorical variables had more than three values Spearman’s rho was applied in the correlations between the instruments’ total score. The p-value was set at 0.05 for all the analyses.

## Results

In our hospital, medical staff consists of 650 workers. As we mentioned above, 110 responders were enrolled, comprising 16.9% of all medical staff working in our hospital. 

The descriptive characteristics of the study sample are presented in Table 1. In general, the majority of the sample consisted of females (60%) and married (61.8%). Half of the participants had received tertiary education (50%). Considerable proportions of the participants were physicians (41.8%) and nurses (45.5%).

**Table 1.** The sociodemographic characteristics of the responders.

Gender	
Males (%)	44 (40)
Females (%)	66 (60)
Age, mean (S.D.)	48.12 (10.30)
Years in service (S.D.)	21.92 (10.65)
Family status	
Married (%)	68 (61.8)
Unmarried (%)	34 (30.9)
Divorced (%)	7 (6.4)
Widowed (%)	1 (0.9)
Number of children (S.D.)	1.87 (0.62)
Educational level	
Primary (%)	1 (0.9)
Secondary (%)	10 (9.1)
Vocational training (%)	2 (1.8)
Tertiary (%)	55 (50)
Post-graduate (%)	32 (29.1)
PhD (%)	10 (9.1)
Occupational status	
Physician (%)	46 (41.8)
Nurse (%)	50 (45.5)
Other (%)	14 (12.7)

*S.D: Standard Deviation, PhD: Doctor of Philosophy

The proportion of clinically significant anxiety and depression is presented in Table 2. As indicated in the table, 38.2% of the study participants had clinically significant anxiety and 6.4% had clinically significant depression.

**Table 2.** The different levels of anxiety and depression in the study sample.

	Absolute values	Percentage (%)
**Anxiety**		
Normal	68	61.8
Clinically significant	42	38.2
**Depression**		
Normal	102	93.6
Clinically significant	7	6.4

As for the effect of the sociodemographic measurements on the score of the instruments, the relevant analysis is presented in Table 3. As indicated in the table, males had lower scores of depression compared to females (p=0.003). In addition, there were significant differences in physical quality of life based on the responders’ occupational status (p=0.019). Yet, not significant differences were found through the post-hoc analysis. As for the effect of educational level on psychological quality of life, the differences were statistically significant (p=0.021). The differences were noted between secondary education participants when compared to tertiary education (Mean Difference -3.527, p=0.021), post-graduate (Mean Difference -3.937, p=0.012) and PhD participants (Mean Difference -5.100, p=0.007). 

**Table 3.** The association of the categorical sociodemographic variables with the instruments’ score.

	Depression		Anxiety		Physical quality of life		Psychological quality of life		Total quality of life	
	Mean (SD)	P	Mean (SD)	P	Mean (SD)	P	Mean (SD)	P	Mean (SD)	P
**Gender**										
Males	35.88 (6.17)	**0.003**	32.90 (6.92)	0.118	20.00 (2.94)	0.186	16.77 (3.07)	0.492	36.77 (4.97)	0.278
Females	39.96 (7.28)		34.87 (7.10)		18.90 (3.75)		16.21 (3.61)		35.12 (6.80)	
**Family status**										
Married	37.52 (6.71)	0.129	33.41 (7.11)	0.052	19.38 (3.40)	0.106	16.55 (3.39)		35.94 (6.00)	0.446
Unmarried	38.76 (8.00)		33.41 (7.11)		19.91 (3.38)		16.44 (3.32)		36.35 (6.03)	
Divorced	44.14 (4.18)		41.42 (5.88)		16.14 (3.62)		15.42 (4.42)		31.57 (7.97)	
Widowed	38.00		33.00		20.00		15.00		35.00	
**Educational level**										
Primary	41.00	0.323	49.00	0.116	13.00	**0.021**	-	0.453	26.00	0.054
Secondary	43.20 (6.82)		38.40 (7.53)		16.00 (3.59)		15.90 (3.24)		31.90 (6.10)	
Vocational training	39.50 (2.12)		31.50 (2.12)		16 (5.65)		14.50 (0.70)		30.50 (6.36)	
Tertiary	37.72 (7.55)		33.85 (6.65)		19.52 (3.34)		16.25 (3.40)		35.78 (6.30)	
Post- graduate	38.25 (6.85)		33.25 (7.78)		19.93 (3.23)		16.71 (3.68)		36.65 (5.90)	
PhD	36.60 (5.46)		32.80 (4.70)		21.10 (1.66)		17.80 (2.93)		38.90 (3.60)	
**Occupational status**										
Physician	37.58 (6.86)	0.597	34.36 (7.59)	0.255	20.38 (2.84)	**0.019**	16.98 (3.32)	0.240	37.76 (5.11)	0.091
Nurse	38.78 (4.44)		33.14 (6.55)		18.17 (3.95)		16.06 (3.65)		34.23 (7.1)	
Other	38.33 (7.12)		36.57 (6.84)		19.34 (3.47)		16.43 (3.40)		35.21 (4.80)	

* PhD: Doctor of Philosophy

The association of numerical sociodemographic variables with the instruments’ score, it is presented in Table 4. As indicated in the table, there were statistically significant associations between age and physical quality of life (r=0.024, p=0.010), years of service and physical quality of life (r=-0.312, p=0.001) and number of children and anxiety (r=-0.027, p=0.002).

**Table 4.** The association of the scale sociodemographic variables with the instruments’ score.

	Depression		Anxiety		Physical quality of life		Psychological quality of life		Quality of life	
	R	P	r	p	r	P	R	P	R	p
**Age**	- 0.016	0.865	0.131	0.174	- 0.245	**0.010**	- 0.027	0.782	- 0.132	0.171
**Years in service**	0.022	0.821	0.154	0.108	- 0.312	**0.001**	- 0.025	0.793	- 0.025	0.793
**Number of children**	- 0.199	0.104	- 0.277	**0.022**	0.022	0.859	0.076	0.537	0.058	0.636

The association between depression and quality of life is presented in Table 5. As indicated in the table, depression had significant inverse associations with both physical and physiological quality and the total score of quality of life (p=0.000). 

**Table 5.** The association between depression and quality of life.

	Physical quality of life		Psychological qualityof life		Quality of life	
	R	P	R	P	r	P
Depression	-0.517	**0.000**	-0.486	**0.000**	-0.575	**0.000**

Finally, the association between anxiety and quality of life is presented in Table 6. As indicated in the table, there was a statistically significant inverse association between anxiety and all aspects of quality of life (p=0.000).

**Table 6.** The association between anxiety and quality of life.

	Physical quality of life		Psychological quality of life		Quality of life	
	R	P	R	P	R	P
Anxiety	-0.529	**0.000**	-0.457	**0.000**	-0.549	**0.000**

## Discussion

This study aimed to investigate depression, anxiety and quality of life in hospital staff of Athens in the aftermath of the debt crisis period. Its results indicate that a high proportion of participants experience clinically significant anxiety (38.2%), but only a small proportion experiences clinically significant depression (6.4%). Depression was higher for females, although in general few associations were noted between sociodemographic variables and the three dependent variables of the study (depression, anxiety and quality of life). Depression and anxiety have an inverse association with quality of life, as well as with its psychological and physical subscales.

Based on the study results, there was a high prevalence of clinically significant anxiety (38.2%) and clinically significant depression (6.4%). Maharaj et al. in their study assessed the status of depression and anxiety in a representative sample of Australian nurses, concluding that depressive and anxiety symptoms were common in nurses with a prevalence rate of over 30% and over 40%, respectively [21]. Carta et al. in their study of depression risk, burnout levels, and quality of life in a sample of workers of an Italian university hospital after the debt crisis period found that the positivity at the screener for Major Depressive Disorder in health care workers is more than double of levels in the standardized community sample. In the same study it was found that all the medical staff, having contact with patients, showed a statistically higher frequency of being screened positive for depressive episode in comparison with the control sample of the population of the region of Sardinia [22]. Gu et al. used an online survey to evaluate mental health problems of 522 healthcare professionals in a Jianghan Fangcang shelter and they reported anxiety and depression with a prevalence rate 25.3% and 51%, respectively [23]. 

A second finding which has to be examined in line with the previous literature regards the higher scores of depression in females. This finding can be attributed to the well-known hormone-related differences, which make females more prone to depression after childhood [24,25], as well as to barriers that females face in workplace, such as sexism [26]. 

Another interesting finding regards the association between Zung’s instruments scoring and quality of life. Even though the association with the mental health component of quality of life is quite expected, the association with physical quality of life is worthy of further investigation. More specifically, increased depression and anxiety lead to elevated stress levels and related physiological effects (e.g., high cortisol levels). These physiological effects lead to deregulation of several systems (e.g., the cardiovascular) or to an increased response of others (e.g., the autoimmune system), finally resulting to physical symptoms and related morbidity [27,28]. Therefore, the association between depression, anxiety and quality of life could be explained by the harmful effects of the stress response on human body. 

Nevertheless, a few limitations for this study have to be reported. At first, the study sample was quite small and not calculated through a relevant formula. Totally, the assessments of the present study were completed by 110 respondents representing 16.9 % of the hospital staff. The sample was small because many of the workers did not want to give information about depression, anxiety and their quality of life. The other reason was the majority of the workers were nurses working in 8-hour shifts and they were not available to respond. Methodologically, this omission can lead to statistical bias, meaning type I or type II error [29]. Thus, it could be realizedthat the study results are prone to this bias. Another limitation of the present research has to do with the cross-sectional design of the study. More specifically, cross-sectional study designs can’t lead to cause-effect associations between two studied variables [30]. Hence, it could be realized that it is unclear if depression and anxiety have a causative effect on quality of life or whether quality of life impacts depression and anxiety. Finally, a major limitation has to do with the generalizability of the study results. Indeed, it is unclear if these results account only for workers of the specific hospital or in general for the hospital staff of public hospitals in Greece.

Based on the results of the present study, a few suggestions for future studies are possible. Firstly, future studies should focus on higher methodological quality, mainly by including larger samples, calculated through relevant formulas. Secondly, the proportion of anxiety and depression found in this study has to be compared with relevant data after the beginning of the pandemic. Indeed, it is worthy of investigation if the levels of anxiety and depression of the hospital staff will increase after the pandemic, taking into account that high rates of mental health problems have already been recorded in hospital staff [13]. Thirdly, several other mental health problems, such as posttraumatic stress disorder (PTSD), are observed in hospital staff [31,32] and have to be investigated in hospitals of Greece.

The results of the study indicate that health policy makers should design interventions to combat health staff anxiety in the National Health System, since it affects a high proportion of workers. Interventions such as psycho-education and cognitive-behavioral training are suitable to decrease anxiety in hospital staff [33] and its implementation in the staff of Greek hospitals has to be considered. In addition, health policy makers have to focus on ways to improve female mental health in hospital workers, since females had higher levels of depression compared to males.

## Conclusions

In conclusion, quality of life was found inversely associated with depression and anxiety, while higher scores of depression were observed in females. Therefore, health policy makers have to reduce this gender gap in mental health between male and female workers. 
